# Quantitative analysis of retinal vessel density and thickness changes in diabetes mellitus evaluated using optical coherence tomography angiography: a cross-sectional study

**DOI:** 10.1186/s12886-021-01988-2

**Published:** 2021-06-15

**Authors:** Xinyue Li, Yu Yu, Xueting Liu, Yan Shi, Xin Jin, Yanyan Zhang, Shuo Xu, Nan Zhang, Li Dong, Sujun Zhou, Yingbin Wang, Yiheng Ding, Zhen Song, Hong Zhang

**Affiliations:** Eye Hospital, The First Affiliated Hospital of Harbin Medical University, Harbin, China

**Keywords:** Diabetic retinopathy, OCTA, Vessel density, Retinal thickness

## Abstract

**Background:**

Diabetic retinopathy is the most common microvascular complication of diabetes; however, early changes in retinal microvessels are difficult to detect clinically, and a patient’s vision may have begun to deteriorate by the time a problem is identified. Optical coherence tomography angiography (OCTA) is an innovative tool for observing capillaries in vivo. The aim of this study was to analyze retinal vessel density and thickness changes in patients with diabetes.

**Methods:**

This was a retrospective, observational cross-sectional study. Between August 2018 and February 2019, we collected OCTA data from healthy participants and diabetics from the First Affiliated Hospital of Harbin Medical University. Analyzed their retinal vessel density and thickness changes.

**Results:**

A total of 97 diabetic patients with diabetes at different severity stages of diabetic retinopathy and 85 controls were involved in the experiment. Diabetic patients exhibited significantly lower retinal VD (particularly in the deep vascular complexes), thickening of the neurosensory retina, and thinning of the retinal pigment epithelium compared with controls. In the control group, nondiabetic retinopathy group and mild diabetic retinopathy group, superficial VD was significantly correlated with retinal thickness (*r* = 0.3886, *P* < 0.0001; *r* = 0.3276, *P* = 0.0019; *r* = 0.4614, *P* = 0.0024, respectively).

**Conclusions:**

Patients with diabetes exhibit ischemia of the retinal capillaries and morphologic changes in vivo prior to vision loss. Therefore, OCTA may be useful as a quantitative method for the early detection of diabetic retinopathy.

**Supplementary Information:**

The online version contains supplementary material available at 10.1186/s12886-021-01988-2.

## Introduction

Diabetes mellitus (DM) is a major cause of death worldwide [1, 2], and the incidence of DM continues to increase [3]. An estimated 415 million adults worldwide had DM in 2015, and the prevalence of DM has been predicted to increase to 642 million by 2040 [4]. With ~ 114 million adults suffering from DM, China is believed to have the highest total number of DM cases [5, 6]. Several factors may contribute to the increasing prevalence of DM in Asia, including lifestyle changes associated with rapid industrialization and urbanization [7, 8].

Diabetic vascular complications are major causes of disability and mortality in diabetic patients [9]. Diabetic retinopathy (DR) is a major microvascular complication of DM and has become one of the leading causes of blindness in China. Approximately 13 million Chinese people ≥45 years of age were affected by DR in 2010 [10]. DR is usually asymptomatic during the early stages of the disease, when changes in retinal microvessels are difficult to detect; however, DR may cause serious vision damage and eventually lead to blindness if not properly treated [11]. Therefore, it is important to identify methods for detecting very small lesions in diabetic retinopathy.

Optical coherence tomography angiography (OCTA) is an innovative tool for examining retinal microvessels in vivo [12] and accurately measuring the thickness of the retinal pigment epithelium (RPE) [13]. Several studies have described the use of OCTA to evaluate DR [13–20]; however, most of these studies have focused on the expansion of the foveal avascular zone (FAZ) and decreases in vessel density (VD), with little attention given to the relationship between VD and retinal thickness. Furthermore, although some studies have documented RPE damage in patients with DM [21–26], in vivo detection of RPE thinning by OCTA has rarely been reported.

The purpose of this study was to investigate retinal microvascular changes using OCTA and explore how the retinal vessel density and thickness of the different layers varied at different DR severities.

## Methods

### Study design and participants

This observational, cross-sectional study was conducted in accordance with the Declaration of Helsinki (1964) and approved by the local clinical research ethics committee (The Ethics Committee of First Affiliated Hospital of Harbin Medical University, registration: ChiCTR1900028128). Healthy participants and patients with Type 2 DM (T2DM) were recruited from the First Affiliated Hospital of Harbin Medical University. All participants signed an informed consent document.

Inclusion criteria for participation in this study were: (1) age ≥ 18 years; (2) intraocular pressure (IOP) ≤ 21 mmHg; (3) visual acuity (VA) < logMAR 1.0; and (4) spherical equivalent (SE) between + 1.00 and − 6.00 D. Exclusion criteria included: (1) other significant eye disease unrelated to DM, including hypertensive retinopathy, retinal vascular occlusion, age-related macular degeneration, or uveitis; (2) major intraocular surgery (including vitrectomy, cataract extraction, scleral buckling, and glaucoma) performed in the past six months or a history of laser photocoagulation or intravitreal injection; (3) glaucoma or first-degree relative with a history of glaucoma; (4) any disease that may cause poor scan quality (image quality < 7), including compact cataract, corneal ulcer, or continuous nystagmus; and (5) macular edema.

### Data collection and grouping

All subjects underwent comprehensive eye examinations, including VA, best corrected visual acuity (BCVA), diopter measurement using automated optometry, IOP measurement using a noncontact tonometer, slit lamp biomicroscopy, and fundus examination. Blood pressure (BP), glycated hemoglobin level (HbA1c), medical history, and family history also were recorded.

DM patients were divided into five groups based on the International Clinical Disease Severity Scale for DR [27]: no apparent DR (no DR), mild nonproliferative DR (NPDR), moderate NPDR, severe NPDR, and proliferative DR (PDR). Healthy subjects without DM were used as a control group.

### Images

We used the FundusVue v2.0.0.3 Ophthalmic Digital Imaging System (Crystalvue Medical Corporation, Taoyuan, Taiwan) to record digital images of each eye through the non-drug dilated pupil. A color fundus photograph obtained during the retinopathy examination was analyzed with one of two plates by a validated retinal expert who followed the Early Treatment of Diabetic Retinopathy Study (ETDRS) guidelines [27].

All OCTA images were obtained using an RTVue imaging device (Optovue, Inc., Fremont, CA, USA). OCTA was used to obtain retinal microvascular images of a 6-mm cube centered on the fovea. For each scan, surface and deep OCTA images were generated based on fully-automated retinal segmentation performed by the OCTA device software. The top and bottom layers of the superficial vascular complexes (SVC) were defined as the inner limiting membrane (ILM) and the inner plexiform layer (IPL) with an offset of 10 μm, respectively. The top and bottom layers of the deep vascular complexes (DVC) were defined as the IPL with an offset of 10 μm and the underlying outer plexiform layer plus Henle’s fiber layer (OPL) with an offset of 10 μm. VD was defined as the proportion of blood flow signal detected by OCTA to the corresponding area (ETDRS Grid projection area unless otherwise specified). The numerical value was calculated automatically by the OCTA software. Thickness of the retinal layers (ILM-IPL, IPL-RPE, ILM-RPE, and RPE-BRM) was automatically measured on the structural map corresponding to the OCTA map obtained simultaneously with the VD map. In addition, the image was divided into superior, inferior, temporal, and nasal parts. The parafoveal area was defined as an annular area 1–3 mm from the foveal area, and the perifoveal area was defined as an annular area 3–6 mm from the foveal area. If there is an error in the recognition of retinal delamination or partition by the machine, the photographer can manually correct it to the correct position.

### Statistical analysis

Baseline characteristics of the various patient groups were summarized and compared using Fisher’s exact tests, analysis of variance, or Kruskal-Wallis tests, depending on the distribution of each variable. Continuous variables were expressed as mean ± standard deviation (SD). Categorical variables were summarized as frequencies and percentages. Spearman rank correlation analysis was used to analyze the relationships of SVC VD and DVC VD with HbA1c, disease duration, mean arterial pressure (MAP = diastolic BP + 1/3*(systolic BP – diastolic BP), and ocular perfusion pressure (OPP = 2/3*MAP – IOP).

Multilevel linear models, adjusted for age, gender, MAP, BCVA, and OPP, were used to analyze the effects of DR severity on VD and thickness of various regions or layers of the macula. We performed a trend test for OCTA across the severity of DR after adjusting for all of the covariables mentioned above. DR severity was further examined as a dichotomous variable by comparing the five groups of DM patients with the healthy control group. The multilevel modelling was used to account for the fact that both eyes were examined from many subjects. Level 1 was each individual eye; Level 2 was the subject. A *P*-value < 0.05 was used to define statistical significance. All analyses were performed using SAS version 9.3 (SAS Institute Inc., Cary, NC, USA), and figures were created using Prism 7.0 (GraphPad, San Diego, CA, USA).

## Results

### Baseline clinical characteristics of study participants

This study evaluated 161 eyes from 97 DM patients and 162 eyes from 85 healthy individuals. The mean age of DM patients was 52.86 ± 11.32 years; 51 patients (53%) were male, 42 patients (43%) were female, and four patients were missing gender information. Among DM patients’s eyes, 87 eyes had no DR, 59 eyes had NPDR, and 15 eyes had PDR. The mean age of healthy individuals was 51.12 ± 13.06 years; 22 (26%) were male and 63 (74%) were female. Demographic and clinical characteristics of all study participants are summarized in Table 1. There were no significant differences in age, MAP, or OPP (all *P* > 0.05; Table 1) between groups. The various groups of DM patients exhibited significant differences in the duration of DM (*P* = 0.0159; Table 1).

**Table 1:** Baseline characteristics of the study participants

Parameter	Control	no DR	Mild NPDR	Moderate NPDR	Severe NPDR	PDR	*P* value*
No. of subjects	85	50	25	7	5	10	
No. of eyes	162	87	41	11	7	15	
Gender^a^							0.0012^†^
Female	63 (74%)	21 (42%)	10 (42%)	2 (33%)	2 (50%)	7 (78%)	
Male	22 (26%)	29 (58%)	14 (58%)	4 (67%)	2 (50%)	2 (22%)	
Age, y	51.12 ± 13.06	51.38 ± 12.13	56.72 ± 9.37	54.71 ± 14.42	44.80 ± 4.55	58.07 ± 9.04	0.656
MAP, mmHg	101.76 ± 8.67	103.03 ± 13.57	105.68 ± 12.90	106.28 ± 13.09	107.08 ± 18.86	101.70 ± 12.91	0.239
VA, logMAR	0.04 ± 0.06	0.15 ± 0.17	0.16 ± 0.17	0.28 ± 0.29	0.55 ± 0.31	0.16 ± 0.12	< 0.0001
OPP, mmHg	52.06 ± 6.47	52.48 ± 9.32	55.35 ± 8.60	55.12 ± 8.61	55.90 ± 11.53	55.40 ± 7.76	0.312
DM duration, m	–	78.38 ± 69.51	138.80 ± 87.17	140.00 ± 106.97	72.06 ± 56.18	168.25 ± 126.46	0.0159
Hypertension	–	20 (40%)	15 (60%)	3 (42%)	2 (40%)	6 (60%)	0.4706^†^
Diabetic nephropathy	–	6 (6%)	2 (8%)	1 (14%)	1 (20%)	1 (10%)	0.4611^†^
HbA1c, %	–	8.42 ± 2.07	8.03 ± 1.53	9.34 ± 0.80	10.04 ± 0	8.56 ± 1.77	0.2477

Data are shown as mean ± standard deviation or *n* (%). ^a^ Four patients were missing gender information. * *P* value: comparison among controls, patients with DM without DR, patients with NDR, and patients with PDR. ^†^ Fisher’s exact test. All patients with systemic hypertension were on antihypertensive medication

### Spearman correlation analysis of associations between VD and MAP, OPP, VA, and HbA1c

Spearman correlation analysis revealed that SVC VD and DVC VD were significantly negatively correlated with the duration of DM (*r* = − 0.1930, *P* = 0.018 and *r* = − 0.2804, *P* = 0.0005, respectively) and with visual acuity (*r* = − 0.2872, *P* = 0.0003 and *r* = − 0.2713, *P* = 0.0007, respectively). However, SVC VD and DVC VD were not significantly correlated with MAP, OPP or HbA1c (Table 2).

**Table 2:** Univariate Spearman correlations between VD^a^ and mean arterial pressure, OPP, VA and HbA1C

Comparison	SVC VD		DVC VD	
	*r* value	*P* value	*r* value	*P* value
MAP, mmHg	0.1414	0.0802	0.0868	0.2844
OPP, mmHg	0.1059	0.1941	0.0525	0.5207
DM duration, m	−0.1930	0.0180	− 0.2804	0.0005
HbA1c, %	−0.1073	0.2072	−0.0926	0.2765
VA, logMAR	−0.2872	0.0003	−0.2713	0.0007

^a^*VD* Whole ETDRS Vessel Density

### Comparison of SCV VD and DVC VD between groups and between retinal regions

**Fig. 1:**
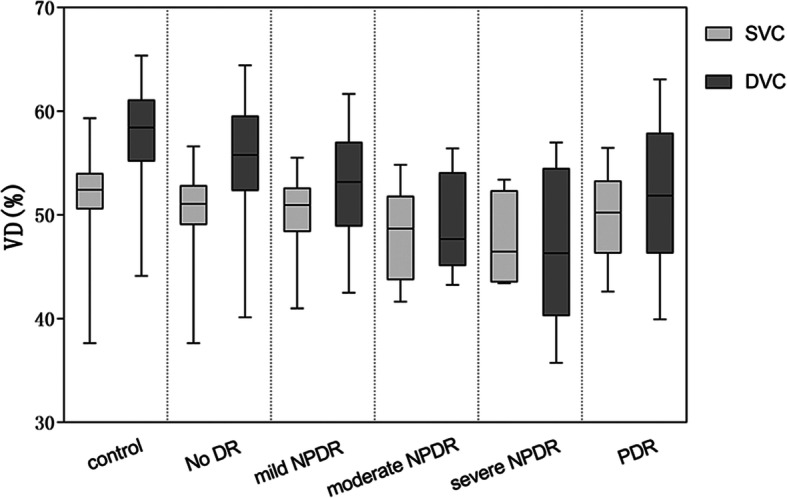
Comparison of SVC VD and DVC VD between groups. Box plots show median, interquartile range, and range. VD in Whole ETDRS regions

**Fig. 2:**
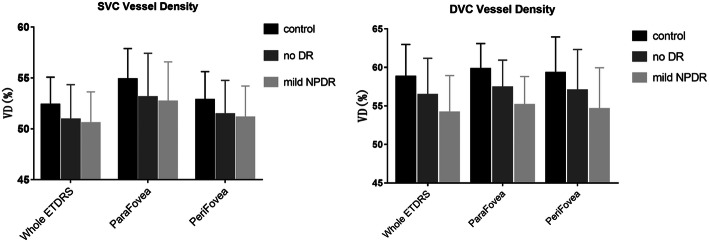
Comparison of SVC VD and DVC VD between the control, no DR, and mild NPDR groups. Data are shown as mean ± SD

**Fig. 3:**
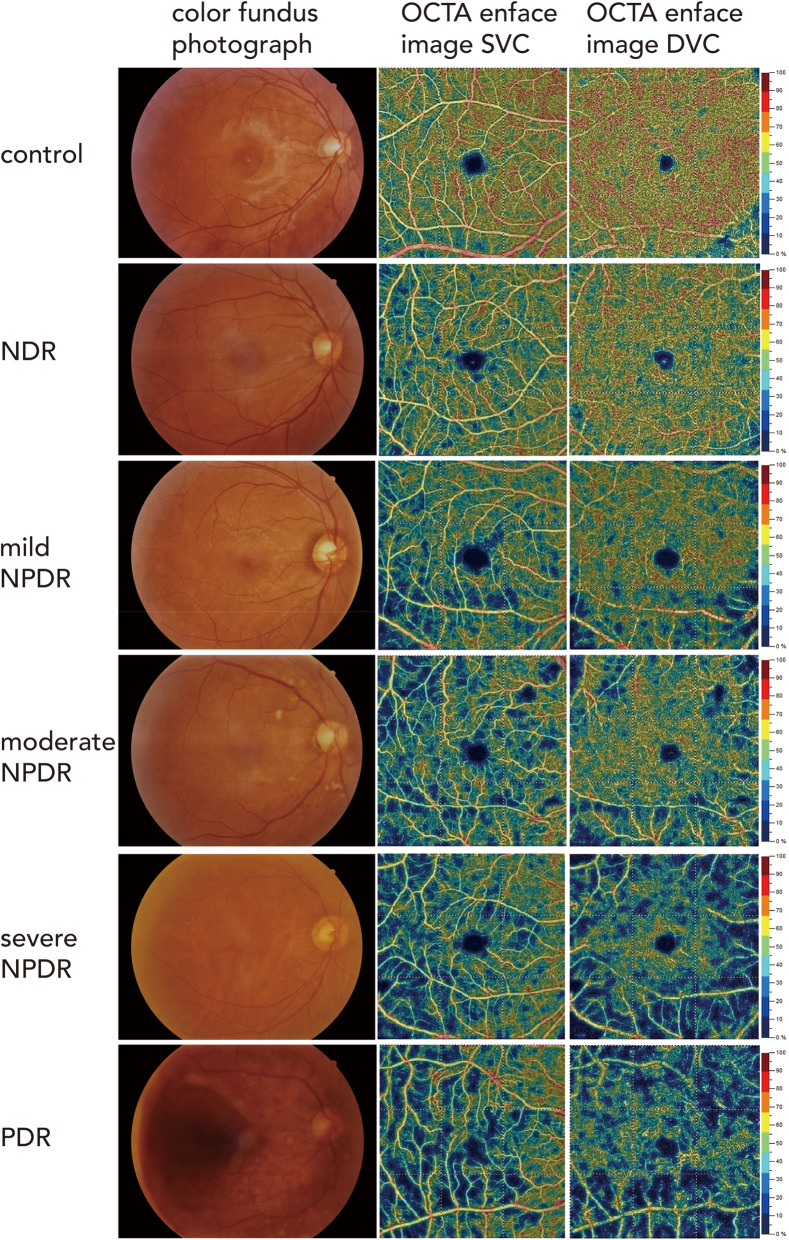
Representative color fundus photographs and OCTA images of the SVC VD and DVC VD in a 6 mm × 6 mm region obtained from control, no DR, mild NPDR, moderate NPDR, severe NPDR, and PDR eyes

**Fig. 4:**
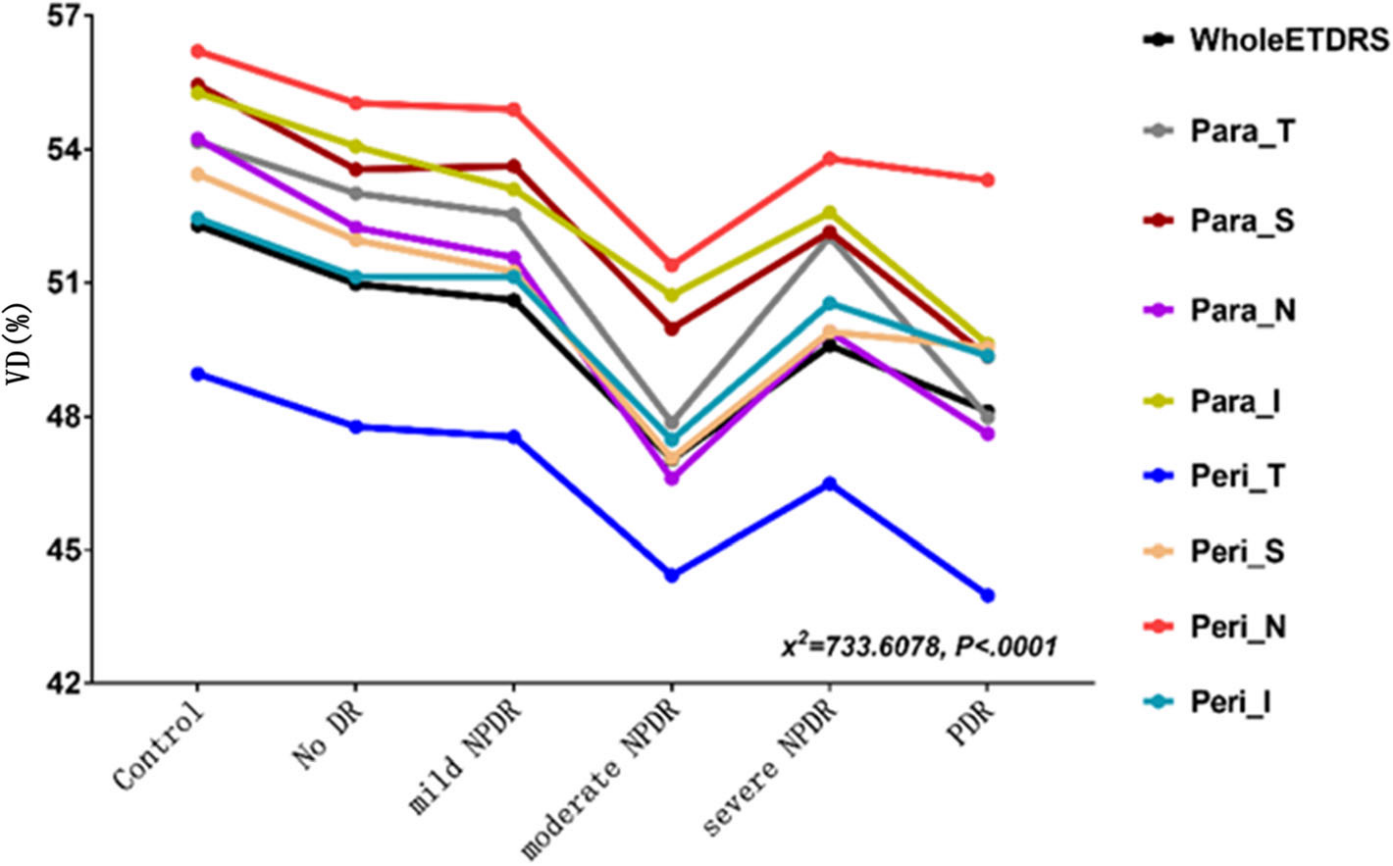
Comparison of SVC VD between different regions of the retina. Para = parafoveal, Peri = perifoveal, S = superior, I = inferior, T = temporal, N = nasal

DVC VD was greater than SVC VD in the control, no DR, and mild NPDR groups but was comparable to SVC VD in the moderate NPDR, severe NPDR, and PDR groups (Fig. 1). Notably, both DVC VD and SVC VD were significantly lower in DM patients than in controls, and there was a significant trend for DVC VD and SVC VD to decrease as the severity of DR increased (Table 3). Interestingly, the reduction in VD was significantly more pronounced in the DVCs than in the SVCs during the early stages of DR (i.e., controls vs no DR vs mild NPDR) in both the parafoveal and perifoveal regions (*P* < 0.001; Fig. 2; Table 3). The differences in VD variation with increasing DR severity between SVCs and DVCs also were evident on the VD images (Fig. 3). Compared with eyes in the control group, eyes in the diabetes groups appeared to contain more dark DVC areas (indicating a lower VD) than SVC areas. Notably, fundus photography did not demonstrate any obvious differences between the control, no DR, and mild NPDR groups. SVC VD was highest in the nasal perifoveal region and lowest in the temporal perifoveal region (*P* < 0.05; Fig. 4; Table 3).

**Table 3:** Mixed effects models showing the correlation between OCTA parameters and the severity of DR

Area	Regression coefficient					
	DR severity ^#^	(Reference = controls)				
		No DR	Mild NPDR	Moderate NPDR	Severe NPDR	PDR
SVC_WholeETDRS	−0.658*	−0.223	− 0.797	− 4.608*	− 2.689	− 2.491
SVC_ParaFovea	− 0.861*	− 0.283	− 1.013	− 4.928*	− 3.851	−3.553
SVC_Para_T	− 0.936*	− 0.222	−0.738	− 5.208*	− 2.806	− 4.310
SVC_Para_S	− 0.664*	− 0.182	− 0.263	− 4.169*	−3.247	− 2.841
SVC_Para_N	− 1.095	− 0.575	− 1.590	− 6.737*	− 5.339	− 4.246
SVC_Para_I	− 0.744*	− 0.036	− 1.306	−3.662*	− 3.263	− 2.976
SVC_PeriFovea	− 0.611*	− 0.191	− 0.691	− 4.593*	−2.376	− 2.254
SVC_Peri_T	−0.781*	− 0.299	− 0.873	−3.799*	− 2.160	−3.746
SVC_Peri_S	− 0.706*	− 0.418	− 1.365	− 5.276*	−3.797	−2.226
SVC_Peri_N	−0.395*	− 0.038	− 0.377	−4.258*	− 2.324	−0.894*
SVC_Peri_I	−0.532*	0.008	−0.079	− 4.976*	− 0.913	− 2.051
DVC_WholeETDRS	−1.382*	0.101	−1.953	−7.022*	−6.664	− 5.482*
DVC_ParaFovea	−1.249*	−0.001	− 1.655	− 6.091*	− 6.828	−4.951*
DVC_Para_T	− 1.209*	−0.213	− 1.727	−5.968*	− 6.406	−4.862*
DVC_Para_S	− 1.203*	0.186	− 0.823	− 5.336*	− 7.629	−4.998*
DVC_Para_N	−1.137*	0.156	−1.415	−7.264*	− 5.280	−4.151*
DVC_Para_I	−1.470*	−0.137	−2.658*	−5.971*	−7.875	− 5.977*
DVC_PeriFovea	−1.468*	0.197	−2.073	−7.388*	−6.775	−5.864*
DVC_Peri_T	−1.320*	−0.034	−1.745*	−5.468*	−5.890	− 5.849*
DVC_Peri_S	−1.536*	0.117	−1.821	−7.664*	−8.176	−6.126*
DVC_Peri_N	−1.517*	0.383	−2.557	−8.744*	−6.965	−5.450
DVC_Peri_I	−1.530*	0.311	−2.249	−7.884*	−5.889	−6.211

Values are adjusted for age, duration, sex, mean arterial pressure, VA and OPP. ^#^ Defined as a continuous variable for the trend test. * *P* < 0.05 vs. control for the same region. *Para* parafoveal, *Peri* perifoveal, *S* superior, *I* inferior, *T* temporal, *N* nasal

### Comparison of perifoveal VD and thickness between groups

**Fig. 5:**
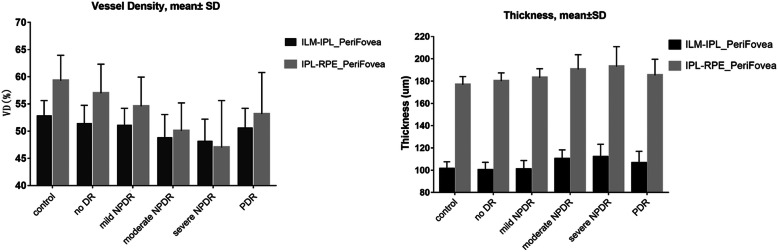
Comparison of perifoveal retinal thickness and VD between groups

**Fig. 6:**
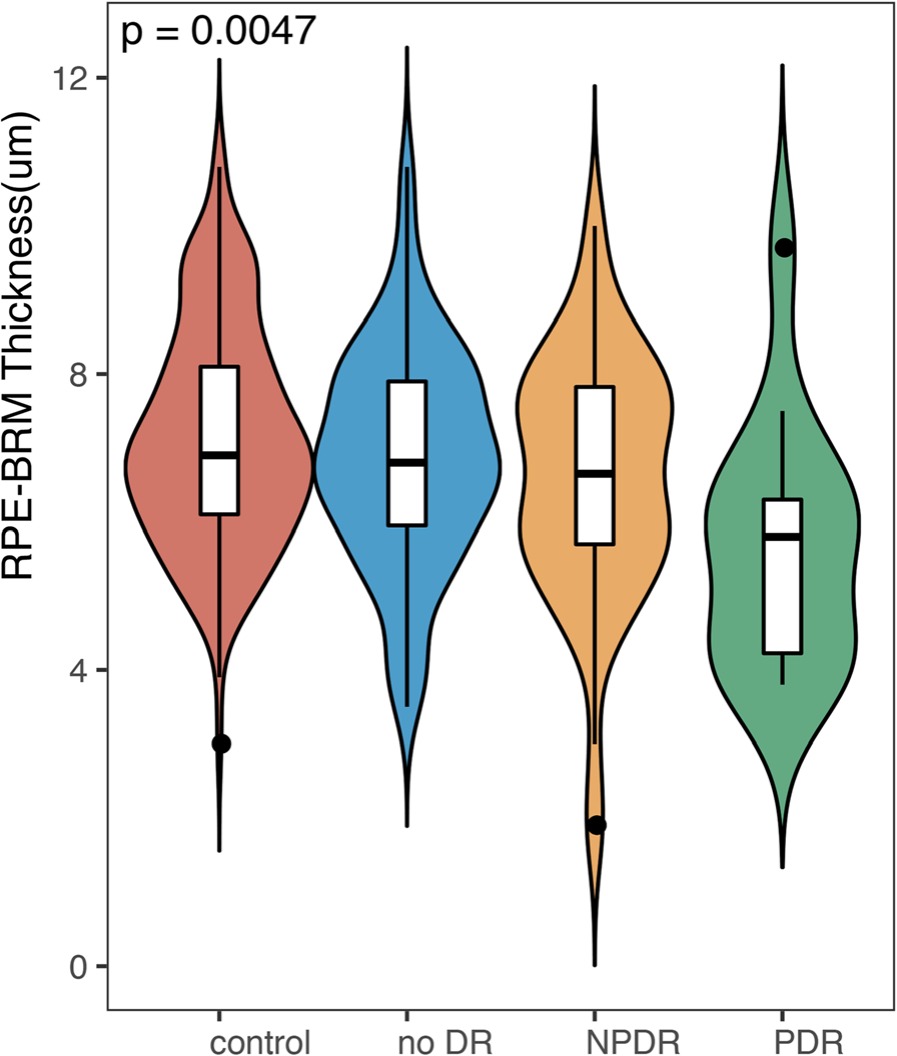
Comparison of RPE-BRM thickness between groups

Perifoveal retinal VD decreased and thickness increased with increasing severity of DR (*P* < 0.05; Fig. 5; Table 4); however, the thickness of the RPE-Bruch’s membrane (RPE-BRM) decreased as the severity of DR increased (*P* < 0.05; Fig. 6; Table 4).

**Table 4:** Mixed effects models showing the correlation between retinal thickness and severity of DR

Area	Regression coefficient					
	DR severity ^#^	(Reference = controls)				
		No DR	Mild NPDR	Moderate NPDR	Severe NPDR	PDR
ILM-IPL Perifovea ALL	1.496*	0.233	0.756	8.423*	17.744*	3.525
ILM-IPL Perifovea T	1.475*	0.329	1.028	5.267	18.243*	4.153
ILM-IPL Perifovea S	1.497*	−2.026	−1.290	11.063*	11.696*	4.275
ILM-IPL Perifovea N	1.690*	1.111	2.058	10.980*	21.001*	3.264
ILM-IPL Perifovea I	1.439*	1.882	2.096	7.767*	22.804*	2.705
IPL-RPE Perifovea All	2.353*	0.857	2.998	7.692*	30.267*	5.826
IPL-RPE Perifovea T	3.434*	1.235	5.105*	14.098*	37.158*	9.216*
IPL-RPE Perifovea S	2.953*	1.040	3.462	6.848	42.838*	8.434*
IPL-RPE Perifovea N	1.422*	0.898	2.256	5.719	19.616*	2.985
IPL-RPE Perifovea I	1.486*	−0.031	1.299	4.169	20.428*	3.622
RPE-BRM Perifovea All	−0.215*	−0.515	−0.376	− 0.661	−1.065	−1.183*
RPE-BRM All	−0.219*	− 0.579	− 0.550	−0.491	−1.273	− 1.173*
ILM-RPE Perifovea All	3.820*	0.977	3.483	15.739*	47.388*	9.064
ILM-RPE Perifovea T	4.905*	1.537	6.075	19.287*	55.265*	13.313*
ILM-RPE Perifovea S	4.415*	−0.894	1.714	17.570*	54.373*	11.768*
ILM-RPE Perifovea N	3.074*	1.892	4.010	16.160*	39.819*	5.958
ILM-RPE Perifovea I	2.922*	1.676	3.200	11.550	42.469*	6.251
ILM-RPE Perifovea All	3.820*	0.977	3.483	15.739*	47.388*	9.064

Values are adjusted for age, mean arterial pressure, sex, duration, VA and OPP. ^#^ Defined as a continuous variable for the trend test. * *P* < 0.05 vs. control for the same region. *S* superior, *I* inferior, *T* temporal, *N* nasal parts

In controls, SVC VD was significantly correlated with the thickness of ILM-IPL (*r* = 0.5090, *P* < 0.0001) and the thickness of ILM-RPE (*r* = 0.3886, *P* < 0.0001) in the perifoveal region. In the no DR and mild NPDR groups of diabetic subjects, SVC VD was significantly correlated with the thickness of ILM-IPL (*r* = 0.4326, *P* < 0.0001; *r* = 0.5237, *P* = 0.0004, respectively) and the thickness of ILM-RPE (*r* = 0.3276, *P* = 0.0019; *r* = 0.4614, *P* = 0.0024, respectively) in the perifoveal. In the moderate NPDR, severe NPDR, and PDR groups, there were no statistically significant associations between SVC VD and perifoveal thickness. However, neither controls nor diabetic patients showed a clear correlation between DVC VD and thickness in the perifoveal (Table 5).

**Table 5:** Comparing the correlation between Vessel Density and Thickness in perifoveal

Comparison^a^	VD SVC Thickness ILM-IPL		VD SVC Thickness ILM-RPE		VD DVC Thickness IPL-RPE		VD DVC Thickness ILM-RPE	
	*r* value	*P* value	*r* value	*P* value	*r* value	*P* value	*r* value	*P* value
Control	**0.509**	**< 0.0001**	**0.3886**	**< 0.0001**	0.0656	0.4039	−0.0381	0.6285
DM	**0.2736**	**0.0004**	**0.164**	**0.0376**	−0.0814	0.3049	−0.1063	0.1798
No DR	**0.4326**	**< 0.0001**	**0.3276**	**0.0019**	0.031	0.7758	0.0684	0.529
Mild NPDR	**0.5237**	**0.0004**	**0.4614**	**0.0024**	0.1828	0.2527	0.2056	0.1972
Moderate NPDR	0.5136	0.1061	0.286	0.3939	0.0182	0.9676	0.0091	0.9892
Severe NPDR	0.4588	0.2529	0.4965	0.2108	0.2664	0.5236	0.4892	0.2186
NPDR	0.216	0.0974	0.0765	0.5611	0.0834	0.5263	0.1813	0.1657
PDR	0.077	0.7937	0.1373	0.6397	0.0745	0.8003	0.0465	0.8746
DR	0.175	0.1358	0.032	0.7866	0.0582	0.6221	0.135	0.2515

^a^ Correlations between various analyzed parameters were calculated using the Pearson test or Spearman rank test, according to the normality of the distribution

## Discussion

An important finding of the present study was that patients in the early stages of diabetes had a lower retinal VD than healthy people even if DR was not present. Retinal VD decreased with increasing severity of DR, especially in the DVC, and was accompanied by thickening of the neurosensory retina and thinning of the RPE. In addition, another important finding of this study was that it directly proved that there was a certain balance between SVC VD and the thickness of the retinal neurosensory layer in the early stage of diabetes. With the aggravation of DR, this balance was broken. Taken together, our results indicated that patients with DM exhibit ischemia of the retinal capillaries and morphologic changes in the retina before DR occurs. Therefore, OCTA may be useful as a quantitative method for detecting early-stage DR.

Our observation that retinal VD was lower in diabetic patients without DR than in healthy controls (Figs. 1, 2, 3 and 4) is consistent with other studies [28, 29]. Notably, three patients with new-onset DM had a lower DVC VD than similarly aged healthy people; whereas, examinations of other target organs revealed no abnormalities. These results suggest that OCTA may be useful for detecting the effects of a high-glucose environment on the ocular microcirculation before detectable abnormalities occur in other target organs. This finding is potentially of great significance for early intervention and timely prevention of large organ damage in patients with DM.

VD also was significantly lower in patients with DR than in healthy individuals (Figs. 2, 3 and 4). The DVC was affected more than the SVC. This apparent difference may potentially help clinicians grade the condition of the retina in early diabetes. Simonett et al. [30] demonstrated that parafoveal DVC VD was lower in patients with T1DM (no DR or mild NPDR) than in controls. Lee and Rosen [16] used OCTA to observe eyes with diabetic macular edema (DME) and found that the FAZ area and microangioma number were higher in the DVC than in the SVC. Similarly, Sambhav et al. [31] found that VD was reduced to a greater extent in the DVC than in the SVC. These findings suggest that a decrease in parafoveal capillary density is an early process in disease progression and occurs initially at the level of the DVC. Several anatomic and physiologic characteristics of the DVC may make it more susceptible to diabetes-induced damage, including greater distance from larger arterioles, higher metabolic requirements close to the external retina, and complex vascular anatomy [32]. In the pathogenic progression of the diabetic retina [33], the first microangioma appears in the deep retina. Interestingly, retinoic acidosis is most prominent in the Outer Nuclear Layer (ONL) in the early stage of a rat DR model, and local acidosis can promote the upregulation of vascular endothelial growth factor (VEGF) and increase the leukocytosis of small retinal capillaries [34]. However, the results of some studies were inconsistent with our findings. Al-Sheikh et al. [35] compared 28 DR eyes with 40 healthy control eyes and found that the SVC VD was significantly reduced, whereas the DVC VD showed no significant changes. Although the reason for this discrepancy is unknown, it should be noted that the Al-Sheikh et al. study had a small sample size, and that it is difficult to accurately assess DVC vasculature using current OCTA without 3D PAR techniques [35]. The flow in more superficial blood vessels may alter the interpretation of deeper blood vessels and explain the differences in the changes in the SVC and DVC [36].

The temporal region of the SVC had the lowest VD and showed the most notable reduction in VD during early-stage DR (Figs. 4, 5 and 6). A histologic study by Kern et al. [37] in an animal model of DM reported that vascular disorders were more prevalent in the superior temporal retina than in the inferior nasal retina. Similarly, Tang et al. [38] showed that diabetic vascular abnormalities occurred more frequently in the temporal retina than in the nasal retina of diabetic human donor eyes. A recent ultrawide field imaging study using Optos devices (Dunfermline, Scotland, UK) also revealed that diabetic vascular abnormalities were more frequent in the temporal fields than in the nasal fields [39]. We speculate that the temporal side of the retinal has a lower VD and increased susceptibility to ischemia and hypoxia because it lies farther away from the disc and has a lower distribution of blood vessels.

Unexpectedly, the VD of PDR patients showed a small but significant upward trend compared with severe NPDR patients (Fig. 1). A possible reason for this finding is that seven of the 15 PDR eyes had no vascular abnormalities in the macula, and any observed vascular abnormalities were primarily microangiomas. OCTA showed blood flow in the microangiomas and recognized intraretinal microvascular abnormalities (IRMAs) as vascular masses with blood flow signals. Careful review of other studies [35, 40, 41] also revealed this phenomenon, although these studies did not offer a specific explanation. Our observations that (1) the duration of disease was negatively correlated with VD and (2) the course of disease in the severe NPDR group was shorter than that in the moderate NPDR group (140.00 ± 106.97 vs 72.06 ± 56.18 months) suggest that the duration of disease may contribute to differences in VD between PDR and severe NPDR patients. The difference caused by the duration of the disease cannot be removed when we drew the figure, but values were adjusted for this duration in Table 3.

Although HbA1c affects microvascular disease and DR in DM patients, SVC VD and DVC VD were not significantly correlated with HbA1c, but were negatively correlated with the duration of DM (Table 2). Similar results were obtained in a previous study [42], which reported that the retinal nerve fiber layer (RNFL) and ganglion cell layer (GCL) were not associated with HbA1c. Although other studies did not observe a correlation between VD and duration of DM [28, 43], these studies were not large enough to account for the duration of diabetes. A 2020 meta-analysis reported that the differences between T1DM groups were nonsignificant; however, these differences became significant for T2DM [44]. The patients in our study were diagnosed with T2DM.

In this study, we observed changes in retinal thickness. The perifoveal retinal thickness increased and the perifoveal retinal VD decreased with increasing DR severity (Fig. 5, Table 4). The primary concern is that RNFL and GCL will become thinner as the severity of DR increases. Chen et al. [45] found that the peripheral inner nuclear layer (INL) and ONL were reduced in patients with T1DM, but were elevated in patients with T2DM.Similarly, Vujosevic et al. [46] demonstrated that the INL was thicker in patients with no DR than in healthy controls and provided evidence that early microglial activation and aggregation may lead to an increase in INL thickness. Bandello et al. [47] also found that increases in retinal thickness are predominantly located in the INL, but may extend to the adjacent retinal layer, possibly due to extracellular fluid accumulation. The INL is mainly formed by the nuclei of bipolar and Müller cells and the combination of horizontal and amacrine cells. Experimental studies [48–50] have reported that activation of Müller cells is accompanied by hypertrophy in the early stages of DR. INL thickening may indicate Müller cell activation that manifests as hypertrophy of these cells. Müller cells are particularly susceptible to hyperglycemia and are considered key factors in the development and progression of retinal damage caused by hyperglycemia [51]. Histopathology analyses have demonstrated that DM is associated with Müller cell proliferation [48]. Due to the important role of Müller cells in regulating the relationship between retinal blood vessels and neurons, metabolic and morphologic changes in Müller cells may induce secondary progressive neuronal loss [52–54]. Prior to the advent of OCTA, in vivo changes in vessel density were difficult to detect. Therefore, few studies have examined the relationship between vessel density and thickness. Dimitrova et al. [28] identified a positive correlation between vessel density and thickness in healthy people, but not in diabetics. Our study observed a positive correlation between SVC VD and thickness in healthy people and found that this correlation also was present in the no DR and mild NPDR groups. No significant correlations were observed in other groups. We hypothesize that under normal circumstances, a proportional relationship exists between vessel density of the retina and thickness of the retina. In the early stages of diabetes, vessels in the retina can automatically regulate mild ischemia. This proportional balance exists when there is decompensated damage of retinal vessels, such as neuronal necrosis (cotton velvet spot) or intraretinal barrier leakage (hard exudation). This proportional balance may be broken with the destruction of the blood-retinal barrier.

An important finding of this study was that high-glucose conditions caused RPE thinning. DM can destroy the blood-retinal barrier [21–25]. The RPE is an important component of the external blood-retinal barrier, and RPE dysfunction is believed to contribute to the development of retinopathy. RPE-mediated loss of blood-retinal barrier integrity is a key feature of DME, a chronic pathology caused by DR. Based on recent studies [22], RPE cells exposed to high glucose levels exhibit structural changes, growth factor/cytokine secretion regulation, and barrier dysfunction [25]. Compared with normal RPE cells, diabetic RPE cells exhibit basal layer thinning, mitochondrial degeneration, nuclear pyknosis, and increased permeability, consistent with the RPE thinning and neuroepithelial thickening observed in the present study. The study of Ponnalagu et al. [25] supported the relationship between RPE and retinal blood flow density at the cellular level and showed that fibroblast growth factor (FGF)-5 expression in the retina and FGF-5 secretion by RPE cells was elevated under diabetic conditions. In patients with NPDR, elevated levels of FGF-1 have been associated with decreased total blood flow to the retina. However, most studies have examined molecular mechanisms underlying high glucose-induced RPE cell injury in vitro, but few investigations have observed RPE dysfunction in vivo. The high-resolution OCTA images obtained in our study suggest that high glucose conditions can cause damage to RPE cells and lead to retinal blood flow changes. Additionally, we observed reduced flow density and thickening of the retinal nerve sensory layer.

This study has several limitations inherent to any study with a limited sample size. For example, patients with macular edema or vitreous hemorrhage were excluded due to poor image quality even though these patients represent the pathogenesis of DR. Exclusion of these patients resulted in a relatively small number of patients in the severe NPDR and PDR groups. However, the no DR group accounted for 54% of the overall disease group, which did not affect our study of patients with early diabetic retinopathy. The gender ratio was significantly different between groups. Therefore, we performed gender-adjusted multiple regression analyses to analyze the effects of DR severity on VD and thickness of various regions or layers of the macula. Coscas et al. [55] reported that VD was higher in women than in men older than 60 years of age, but there was no difference between the genders in other age groups. Because the mean age of all subjects (patients and controls) was 51.98 ± 12.43 years in our study, the results may be less affected by gender. In separate study, Yu et al. [56] showed that while the parafoveal flow index and vessel area density were not associated with gender, the Capillary-free zone was larger in healthy females than in healthy males. Other studies have observed that only FAZ areas were influenced by gender, rather than VD [57, 58].

## Conclusions

OCTA is a valuable tool that produces unique high-resolution images of retinal capillaries. Rapid noninvasive OCTA was useful to quantify retinal microvascular abnormalities, RPE cell atrophy, and retinal neurosensory layer thickening in vivo in DM patients who did not have clinically detectable retinopathy. Therefore, OCTA may be a valuable quantitative method for detecting early-stage DR.

## Supplementary Information

**Additional file 1.** (AVI 13520 kb)

**Additional file 2.**

**Additional file 3.**

**Additional file 4.**

## Data Availability

The datasets generated and analyzed during the current study are not available due to ethical restrictions and ongoing studies but are available from the corresponding author on reasonable request.

## Abbreviations

OCTA: Optical coherence tomography angiography
DM: Diabetes mellitus
FAZ: Foveal avascular zone
VD: Vessel density
VA: Visual acuity
BCVA: Best corrected visual acuity
IOP: Intraocular pressure
mild NPDR: Mild nonproliferative DR
PDR: Proliferative DR
SVCs: Superficial vascular complexes
ILM: Inner limiting membrane
IPL: Inner plexiform layer
DVCs: Deep vascular complexes
OPL: outer plexiform layer plus Henle’s fiber layer
SD: Standard deviation
IRMAs: Intraretinal microvascular abnormalities
VEGF: Vascular endothelial growth factor
RNFL: Retinal nerve fiber layer
GCL: Ganglion cell layer
INL: Inner nuclear layer
FGF: Fibroblast growth factor
